# Ocular Defects in Photosensitive Epilepsy

**DOI:** 10.1100/tsw.2004.21

**Published:** 2004-03-12

**Authors:** Ebere C. Anyanwu, John Ehiri

**Affiliations:** ^1^ Cahers Neurosciences Research Inc., 8787 Shenandoah Park Drive, Suite 122, Conroe, TX, USA; ^2^ Department of Maternal and Child Health, School of Public Health, University of Alabama at Birmingham (UAB), Ryals Public Health Building, 1665 University Boulevard, Birmingham, AL 35294-0022, USA

**Keywords:** photosensitive epilepsy, visual-evoked potential, ocular defects, electroencephalogram

## Abstract

Patients with photosensitive epilepsy are susceptible to seizures due to photoparoxysmal response (PPR). This response adversely precipitates factors that modify the functional status of the visual system. Such factors may or may not be evident superficially, but may lead to ocular defects due to trauma, hormonal imbalance, abnormal intraocular pressure (IOP), or any other reflex-inducing stimuli. The extent to which photosensitive epileptic patients suffer from PPR-related ocular defects has not been documented fully. In this investigation, ocular defects in patients with photosensitive epilepsy are studied using visual-evoked response (VER). A total of 212 photosensitive epileptic patients were studied to ascertain the magnitude and distribution of ocular defects using the changes in EEG and visual-evoked potential (VEP); 51% of the patients were female, the age range was 1—46 years. The major ocular defects and complications found were visual field defects, optic nerve abnormalities, nystagmus, cataracts, amblyopia, and migraine. These findings were analyzed according to age and sex. The relationship between the ocular abnormalities and the interpretations of the changes in the characteristics of the VEP indicated that optic-related atrophies, visual defects, optic neuritis, chiasmal compression, nystagmus, migraine headache, cataracts, and amblyopia were prevalent in photosensitive epileptic patients at varying degrees. The results showed that although ocular defects in photosensitive epilepsy may not be obvious differentially, VEP can be used in their diagnosis, contrary to earlier studies reporting that VEP is not of much value in epilepsy diagnosis.

## INTRODUCTION

Patients with photosensitive epilepsy are susceptible to seizures due to photoparoxysmal response (PPR). This response adversely precipitates factors that modify the functional status of the visual system. Such factors may or may not be evident superficially, but may lead to ocular defects due to trauma, hormonal imbalance, abnormal intraocular pressure (IOP), or any other reflex-inducing stimuli. The application of visual-evoked potential (VEP) in the diagnosis of ocular defects in photosensitive epilepsy has not received much attention, because it is believed that VEP testing is not of much value in epilepsy diagnosis[1]. Early papers reported "activation" of the VEP during seizures[2,3,4], but these reports were not specific and often offered contradictory results. Subsequent papers provided little substantive information, although one report increased VEP amplitudes in patients with photoconvulsive seizures[5]. The usefulness of VEP in neuro-ophthalmic disorders has been described[6] and can be applied effectively to photosensitive epilepsy. VEP can be used to locate lesions at specific levels of the visual system, either to confirm or exclude various neuro-ophthalmic diagnoses. VEPs, since they originate mainly in the cortex, may be affected by lesions affecting the visual pathway at any level[6]. Therefore, lesions that occur at ocular, optic nerve, chiasmal, optic tract, or cortical levels can affect the response. By using pattern stimulation or flash monocularly, it is possible to differentiate pre- and postchiasmal lesions, as can be done with more stimuli (such as half field stimulation)[6].

Salanova et al.[7] reported the result of ocular findings in 42 patients with medically refractory occipital lobe epilepsy who underwent surgery at a neurological institute between 1930 and 1991. They found that in more than two-thirds of the patients, the clinical ocular manifestations indicated an occipital onset of seizures. Of the patients, 73% experienced visual auras, of which elementary hallucinations were the most common; 12 also had ictal blindness. Other occipital manifestations included contralateral eye deviation, blinking, a sensation of eye movement, and nystagmoid eye movements. Cortical stimulation elicited a habitual aura in 37% of 29 patients. Lateralizing clinical features were seen in almost two-thirds of the patients, contralateral head deviation occurred in half, 59% had visual defects contralateral to epileptogenic area, and 64% had abnormal image manifestations ipsilateral to the side of surgery.

In this study, results are presented for 212 patients whose diagnoses were based on abnormalities derived from VEPs.

## METHODS

Patients with photosensitive epilepsy are susceptible to seizures due to PPR, which may adversely affect the functional status of the visual system. The extent to which these adverse ocular defects are manifested are not known fully. Hence, a retrospective study was carried out on a total of 212 photosensitive epileptic patients to assess the magnitude of these ocular defects that may or may not be evident superficially.

All the patients had a basic EEG including hyperventilation recorded on 16-channel Elema Schonander SLE machine using the International 10–20 electrode placement system. A parasagittal montage was always used during photic stimulation at light intensity 2 with pattern grid using the 2-sec timer[8]. The patients were tested with their eyes either open or closed. The room light standard was artificial light only. The lamp with grid was placed 30 cm from the face of the patients and the patients were asked to fixate the encircled area at the center of the lamp.

VEP measurements were carried out and from these test procedures, the changes in the VEPs recorded, and those changes that represented ocular defects were identified. The patients were then grouped and analyzed according to sex, age, and the type of ocular defects.

## RESULTS

Out of a total of 212 photosensitive epileptic patients studied, 104 (49%) were males and 108 (51%) were females. The ages ranged between 1–46 years. The prevalent ocular defects found in this population were optic-related atrophies, visual field defects, optic neuritis, chiasmal compression, nystagmus, migraine headache, cataracts, and amblyopia (Table 1). Optic-related atrophies, including cortical atrophy (33%), had the highest rate of incidence and occurred in 16% of females and 17% of males (M/F = 1:1 ratio). Of the study population, 27% had visual field defects (males = 12%, females = 15%, giving a male/female ratio of 1:1). The ocular defects with the least occurrence were nyastagmus (7%), migraine (6%), and amblyopia (5%).

**TABLE 1**
**Sex Distribution of Ocular Defects in Photosensitive Epilepsy**

| | 1 | 2 | 3 | 4 | 5 | 6 | 7 | 8 |
|---|---|---|---|---|---|---|---|---|
| **Sex** | **Opt. A** | **FLD** | **Opt. N** | **ChComp** | **Nyst** | **Migr** | **Cat** | **Ambl** |
| Male | 35 | 26 | 13 | 6 | 10 | 6 | 2 | 6 |
| Female | 34 | 32 | 12 | 11 | 5 | 6 | 4 | 4 |
| Total | 69 | 58 | 25 | 17 | 15 | 12 | 6 | 10 |

**Key:** Opt. A = optic-related atrophy; FLD = field defects; Opt. N = optic neuritis; ChComp = chiasmal compression; Nyst = nystagmus; Migr = migraine; Cat = cataract; Ambl = amblyopia.

It was observed that most of the ocular defects in photosensitive epilepsy were distributed evenly across the sexes, except that there were some significant differences in the occurrence of chiasmal compression (M/F ratio = 1:2) and cataracts (M/F ratio = 1:2). In amblyopia and nystagmus, the M/F ratio was 2:1, respectively. Analysis of age of distribution of defects showed that most susceptible age range for ocular defects in photosensitive epilepsy is 6–10 years (32%), while the least susceptible age range is over 45 years (Table 2).

**TABLE 2**
**Age Distribution of Ocular Defects in Photosensitive Epilepsy**

| Age | Number of Patients | % |
|---|---|---|
| 5 | 22 | 10 |
| 6–10 | 68 | 32 |
| 11–15 | 41 | 19 |
| 16–20 | 36 | 17 |
| 21–25 | 17 | 8 |
| 26–30 | 13 | 6 |
| 31–35 | 7 | 3 |
| 36–40 | 5 | 2 |
| 41–45 | 2 | 1 |
| >46 | 1 | 1 |
| Total | 212 | 100 |

## DISCUSSION

### Visual Field Defects

The recognition of ocular defects in photosensitive epilepsy using visual-evoked response (VER) depends greatly on a thorough understanding of the differences between abnormal and normal VERs. This requires a clear knowledge of the morphological and temporal characteristics of the VER and the pathophysiolocal mechanisms that underlie the abnormalities. It is not clear whether the sensitivity of VER in photosensitive epilepsy is exclusively in response to lesions in the visual pathway, in the visual cortex, or both. However, it is well accepted that any abnormalities in VER may represent a defect in the visual system. The validity of this claim was demonstrated by Halliday et al.[9], who recorded the response to a centrally fixated screen of wide-field (WF) stimulus at a single occipital electrode. This simple method identifies the major surface-positive wave of WF response (P100) as an indicator of an abnormality. Clinically, VER is now regarded as a single positive wave[10].

The electrophysiological basis of VER tests is the comparison between the changes in the latency or amplitude of P100 components observed in both normal and abnormal subjects[10]. The evoked response is related markedly to the amount of cortical neurons involved and obviously influenced by any macular defects[10]. Although there are many published papers on this topic, they are based on single cases or have methods that present difficulties in interpretation or seem to have given equivocal results. The type of macular degeneration is not always specified and none are described as decimet form. Studies limited to patients with established decimet degeneration, particularly unilateral disease[10], showed that the VER from the stimulated normal eye was similar to that with binocular stimulation and was acceptably normal for the age of the patient. When the affected eye was stimulated, however, the P2 wave was markedly reduced[11]. In the case of bilateral decimet degeneration, the response was worse in the affected eye; there was a preserved P1 and hardly any P2. These results suggest that P2 wave is related to macular function because it is reduced when the central area of the fundus is protected from the stimulating flashes of light by occlusion[11]. Phase reversal techniques are also shown to be a clear indicator of any asymmetry of the decimet degeneration. Phase reversal is obtained only from the visual cortex related to the preserved retina. A degenerated retina is indicated by reduced amplitude, delayed latency, and absence of components of the evoked response[10,11].

### Optic Atrophy

Optic atrophy is impairment of the optic nerve due to the degeneration of the nerve fibers and optic tract. It is characterized by pallor of the disc, a whitish, yellowish, or grayish appearance. It is accompanied by visual field loss. The condition can be due to a variety of factors originating from epileptogenic activities in the central nervous system (CNS). The underlying innate or acquired predisposition to seizures, plus a precipitating factor that determines the appearance of seizures at a particular point in time, adversely modify the functional status of the CNS. Such factors may or may not be evident and may include injury as a result of infection, hormanal imbalance, photic stimulation, abnormal intercranial pressure (ICP), or any other reflex-inducing stimuli[12]. For example, increase in ICP can be caused by generalized acute reversible or chronic venous compression, out-flow obstruction, and generalized seizures[13]. Some of the symptoms observed in the current study include confusion, restlessness, blurring vision, diplopia due to abducens paresis, headache, and various motor anomalies. Apart from optic atrophy accompanying glaucoma, the etiology and the mechanism of optic atrophy in the study population is not specific. However, the study has shown a 16–17% frequency of occurrence in the photosensitive patient sample, with a sex ratio of 1:1. In photosensitive epilepsy, it is assumed that optic atrophy is caused at least in part by the frequent assault on the optic nerve and tracts during epileptogenic activities.

## Optic Neuritis

Optic neuritis is one of the clinical "syndromes" observed in this study. The signs and symptoms of disease are variable: unilateral blurring of vision, ocular pain, paracentral scotoma, attacks of double vision, trigeminal neuralgia, and sometimes intentional tremor. Extraocular muscles may be affected, resulting in the disturbances of conjugate movement, gaze palsy, horizontal, mixed or vertical nystagmus, strabismus, and abnormal light-blink reflex.

Optic neuritis made up 12% of photosensitive epileptic patients in the study population. It was found mostly in the adult population, aged 21 years and above. In most cases of optic neuritis, there are delays of WF responses in the range of 10–100 msec, with mean values of 30–40 msec. Also, as a result of the presence of demyelination, ipsilateral half-field (HF) components of the VEP appear to show differential latency changes, with earlier waves (e.g., N75 and the "leading descending limb" of the P100) showing less marked delays then later potentials (example, the "trailing ascending limb" of the P100 and the N145 wave)[13]. Many studies linking VER with optic neuritis are inconclusive. Some authors have reported a significant variation in VER of some patients and suggested that there is little or no change in amplitude[14], while others reported that the latency of P2 component of VER showed consistent delay in unilateral optic neuritis[9]. It has been shown that pattern reversal stimulation is an effective diagnostic technique in identifying delays in VEP due to demyelination. This effectiveness has been confirmed by subsequent studies[15,16,17]; although many studies used 50-min check standards with a variation of 30 and 70 min in pattern reversal. It is suggested[18,19] that such a variation was not critical except in studies of acute neuritis in the florid stage, when smaller checks cannot be seen and therefore do not elicit a response.

## Chiasmal Compression

Chiasmal compression was observed in 8% of the patients in the current study. The mechanism of abnormal response in chiasmal compression is based on the amplitude asymmetries involving delays in WF and HF. The major involvement of the decussating fibers arising from the nasal retina results in an asymmetry, which characteristically changes sides of the scalp with monocular stimulation of the eye[13]. Stimulation by pattern or flash is usually performed monocularly in order to allow prechiasmal differentiation. It has been shown that the sensitivity of WF in detecting prechiasmal lesions in the anterior visual pathways involves a wide range of diseases, including photosensitive epilepsy[16,17]. But for postchiasmal lesions, multichannel recording from both cerebral hemispheres, using more specific stimuli such as HF stimulation, is effective[17,18,19]. Overall, the combination of monocular stimulation and multichannel recording allows identification of chiasmal lesions[6]. Although these pathologies are rarely symmetrical in practice, the evidence of waveform distortion is great. If the posterior angle of the chiasma is involved in a lesion, there is always the introduction of a homonymous element, and the anterior compression consequently will involve one optic nerve, resulting in wave form distortion, attenuation, or delays. The relationship between chiasmal compression and other visual field defects in photosensitive epilepsy, with respect to changes in VER, remains to be clarified fully.

## Nystagmus

Nystagmus is an ocular defect that is characterized by repeated "to and fro" oscillations of the eye. It is associated with the neurological diseases of the visual and vestibular systems, hence it is described as vestibular, optokinetic, or endpoint nystagmus[17,19,20]. It can be congenital in origin, in which case the onset is within the first decade of life. Congenital nystagmus is due to metabolic disorders, hypothyroidism, or structural brain anomalies[17,19,20,21]. Nystagmus eye movement can be found in albino and photosensitive epileptic patients, in aniridia patients, patients with achromatopsia (no cones or limited number of cones which result in rod vision), and congenital cataract patients. Typically, nystagmus is horizontal (predominantly pendular in presentation) or jerky (in which case the eyes slowly drift away from the fovea and then quickly jerk back towards the fovea). Nystagmus was present in about 7% of the population in the current study and characterized electrophysiologically by an amplitude of 4° at a frequency of 3–4 Hz.

## Migraine Headaches

Migraine headache is characterized by a syndrome of periodic headache that may become generalized. Patients may display some degree of irritability and nausea often with photophobia, vomiting, constipation, and diarrhea[20,21,22]. Frequently, the attacks may be heralded by scotomata, hemianopia, unilateral paraesthesias, and speech disorders[20]. The pain is commonly limited to the head, but may include the face, the ocular adnesxia, and even the neck. It may be throbbing, gradual in onset, and may last several hours to several days[21]. Migraine headache was observed in 6% of the study population.

## Cataract

The application of VEP in the prognosis of cataracts has been very slow, probably because of the opacity of the media. Nevertheless, Arden and Sheorey[23] demonstrated techniques that provided pattern stimulation through opaque media. Earlier studies by Copenhaver and Perry[24] revealed a reduction in flash VEP in patients with opacities, but these results had no correlation with visual acuity. In the current study, only 3% of photosensitive epilepsy patients, of which the ratio between male and female was 2:1, respectively, had cataracts. Out of this number, more than 50% had visual acuities of 20/40 (6/12) or worse.

## Amblyopia

Amblyopia was found in 5% of photosensitive epileptic patients in the study, with the incidence more in males than females by a ratio of 2:1. Uncorrected visual acuities 20/40 (6/12) or worse were found in more than 40% of these patients. One of the patients had strabismus, with a visual acuity of 20/80 (6/24) in both eyes. The reason for this observation was not ascertained readily, however, it could be due to a refractive amblyopia affecting each eye. Earlier comprehensive studies[24] on VER as a possible method for the diagnosis of visual defects, particularly amblyopia, was carried out on both "normal eyes" and affected amblyopia eyes. It was found that response to foveal unpatterned flash stimulus was reduced in amplitude, but the responses to stimuli of 20° field was not reduced[25].

The most consistent finding in the current study was that with foveal stimulation, using small targets, there was a reduction in amplitude and increase in latency from the stimulation of the amblyopic eye irrespective of the color of the light[25,26,27]. Although the type of amblyopia was not specified for individual patients, there was a consistent reduction of VER and hence a reduction in amplitude in all cases of amblyopia. It was also observed consistently that patients with amblyopia showed a general stimulation and pattern stimulation consistent with the results reported by Harding[28]. The maximum evoked potential amplitude from stimulation of the amblyopic eye could be obtained at larger check sizes than for the normal eye[29]. Although this would be expected by the nature of the condition[30], in some amblyopic patients the interocular difference may exceed the normal limits of 27°, whereas binocular summation in normals increased amplitude by 12°[30]. This increase in amplitude was either not achieved or, in some cases, was reduced in amplitude[6].

## CONCLUSION

The ocular defects in photosensitive epilepsy have been investigated. Since patients with photosensitive epilepsy are susceptible to seizures due to luminance variance, it is obvious that the cells in the visual system of such patients may respond adversely to the stimuli that have the propensity to modify the functional status of the visual system. Such changes may lead to abnormalities in ocular structures and consequently affect the clarity of vision. The outcomes of this investigation showed clearly that although ocular defects in photosensitive epilepsy may not be obvious differentially, however, VEP measurements can be used in their diagnosis, contrary to earlier studies that stated that VEP was not of much value in epilepsy diagnosis.

## BIOSKETCHES

**Ebere C. Anyanwu, PhD,** is a Clinical Neurophysiologist and Director of Cahers Neurosciences Research, Inc., Conroe, TX. He is also an Adjunct Professor of Anatomy and Physiology at the North Harris and Montgomery Colleges in Houston and The Woodlands, respectively. Also a Researcher at the Biomarkers Research Laboratory, Department of Environmental Chemistry and Toxicology, Texas Southern University, Houston. E-mail: ebereanyanwu@msn.com

**John Ehiri, PhD, MPH,** is an Assistant Professor in the Department of Maternal and Child Health, School of Public Health, University of Alabama at Birmingham (UAB), Birmingham, AL. E-mail: jehiri@uab.edu.
